# Association of the maternal fish and PUFA intake with the risk of hypertensive disorders of pregnancy: a nationwide birth cohort—The Japan Environment and Children’s Study (JECS)

**DOI:** 10.1007/s00394-026-04103-7

**Published:** 2026-09-08

**Authors:** Takuya Inouye, Daisuke Higeta, Kei Hamazaki, Akira Iwase, Akiko Tsuchida, Hidekuni Inadera, Eiji Yoshioka, The Japan Environment and Children’s Study Group

**Affiliations:** 1 Department of Obstetrics and Gynecology, Gunma University Graduate School of Medicine, Gunma, Japan; 2 Department of Public Health Faculty of Medicine, Gunma University Graduate School of Medicine, Showa 3 Maebashi, Gunma, Japan; 3 Department of Public Health, Faculty of Medicine, University of Toyama, Toyama, Japan; 4 Toyama Regional Center for JECS, University of Toyama, Toyama, Japan

**Keywords:** Birth cohort, Fish, Polyunsaturated fatty acids, Hypertensive disorder of pregnancy, Preconception care

## Abstract

**Purpose:**

Hypertensive disorders of pregnancy (HDP) are a leading cause of maternal and perinatal morbidity. Although n-3 polyunsaturated fatty acids (PUFAs) in fish may prevent HDP, evidence from Japanese populations, particularly regarding the preconception period, remains limited. To examine associations between fish and PUFA intake before and during pregnancy and the risk of HDP using data from a nationwide Japanese birth cohort.

**Methods:**

This analysis included 86,009 women from the Japan Environment and Children’s Study. Dietary fish and n-3/n-6 PUFAs intake was assessed using a validated food-frequency questionnaire administered in early pregnancy (median gestational age, 15.0 weeks [interquartile range (IQR) 12.0–19.0]) to capture intake before pregnancy, and in mid to late pregnancy (median gestational age, 27.0 weeks [IQR 25.0–30.0]). Participants were categorized into quintiles of intake. Logistic regression models were used to estimate adjusted odds ratios (aORs) and 95% confidence intervals (CIs) for HDP, early-onset HDP (Eo-HDP < 32 weeks), and late-onset HDP (Lo-HDP ≥ 32 weeks). Missing data were addressed with multiple imputation.

**Results:**

In the overall population, higher fish intake before pregnancy was associated with reduced risks of Eo-HDP in the Q5 groups, whereas higher fish intake during pregnancy was associated with reduced risks of overall HDP in Q4. In analyses restricted to nulliparous women, higher pre-pregnancy fish intake was associated with a lower risk of Eo-HDP (Q4: aOR 0.57; 95% CI 0.34–0.97; *p* for trend = 0.02), and higher fish intake during pregnancy was associated with a lower risk of Lo-HDP (Q4: aOR 0.79; 95% CI 0.64–0.96). Total n-3 PUFA intake showed significant associations with HDP only in some analyses. Sensitivity analyses using a 34-week cutoff yielded consistent results.

**Conclusion:**

Fish intake before pregnancy was associated with lower risk of Eo-HDP, whereas intake during pregnancy was linked to reduced risk of Lo-HDP. Sustained fish consumption from preconception through pregnancy may help prevent HDP and inform global nutritional guidance, including preconception care.

**Trial registration:**

The Japan Environment and Children’s Study, https://upload.umin.ac.jp/cgi-open-bin/ctr_e/ctr_view.cgi?recptno=R000035091 (Registration no. UMIN000030786).

**Supplementary Information:**

The online version contains supplementary material available at https://doi.org/10.1007/s00394-026-04103-7.

## Introduction

Hypertensive disorders of pregnancy (HDP) are a group of conditions characterized by new-onset hypertension during pregnancy, including preeclampsia (PE), gestational hypertension (GH), and chronic hypertension. HDP occur in approximately 5–10% of all pregnancies [1] and they remain among the leading causes of maternal and perinatal morbidity and mortality worldwide [2, 3]. Established maternal and cardiometabolic risk factors for HDP, such as obesity, diabetes, and socioeconomic factors, have been well documented [4]. Indeed, HDP is a multifactorial disorder, and sufficient evidence has accumulated regarding the factors and related determinants involved in its development. In addition, women who develop HDP are at increased risk of long-term cardiometabolic diseases later in life [5–9]. Therefore, preventive strategies focusing on long-term maternal health are essential.

Evidence from observational studies suggests that associations between dietary fatty acid intake and the risk of HDP differ according to the type of fatty acid consumed [10–14]. N-3 polyunsaturated fatty acids (PUFAs), found abundantly in fish and shellfish, possess anti-inflammatory and vasodilatory properties and may have protective effects against HDP. Previous observational studies, including two prospective cohort studies and one case-control study, have mainly examined individual n-3 PUFA subtypes [15–17]. Furthermore, while a large prospective cohort study reported an inverse association between higher fish intake during pregnancy and the risk of HDP, an earlier case-control study did not observe a significant association [18, 19]. In addition, low-dose aspirin (LDA) has been shown to be effective in addressing the pathophysiology of HDP, which involves impaired spiral artery remodeling [20, 21]. Given the anticoagulant properties of n-3 PUFAs, similar beneficial effects may be anticipated [22, 23].

Recently, the concept of preconception care has gained increasing attention [24]. Maternal and child health may be influenced not only during pregnancy but also by nutritional status before conception. For example, preconception dietary patterns have been associated with the risk of HDP [25–28].

Most studies examining associations of fish or fatty acid intake with HDP have focused on maternal diet during pregnancy, mainly in Western populations [10–16, 18, 19]. Findings regarding the association between fish intake during pregnancy and HDP have also been inconsistent. In Japan, where dietary habits differ considerably, epidemiological studies on the relationship between preconception fatty acid intake and HDP are limited. Although fish consumption has traditionally been high in Japan, recent reports have indicated a decline in intake among younger women [29]. Therefore, examining the frequency of fish and seafood intake within the Japanese diet is essential.

This study aimed to clarify the associations of fish and n-3 PUFA intake before and during pregnancy with the risk of HDP using data from the Japan Environment and Children’s Study (JECS), a nationwide birth cohort. Our findings aim to provide scientific evidence to support nutritional interventions in maternal health policy, including preconception care.

## Methods

### Study population

The JECS protocol has been described in detail elsewhere [30, 31]. Briefly, the JECS was designed to evaluate the effects of specific environmental factors on child health and development. Pregnant participants were recruited between January 2011 and March 2014 from 15 Regional Centers across Japan [30, 31]; the median gestational age at recruitment was 12.0 weeks (interquartile range [IQR], 10.0–15.0 weeks). The present study was based on data obtained from the JECS, an ongoing nationwide prospective birth cohort study.

**Fig. 1:**
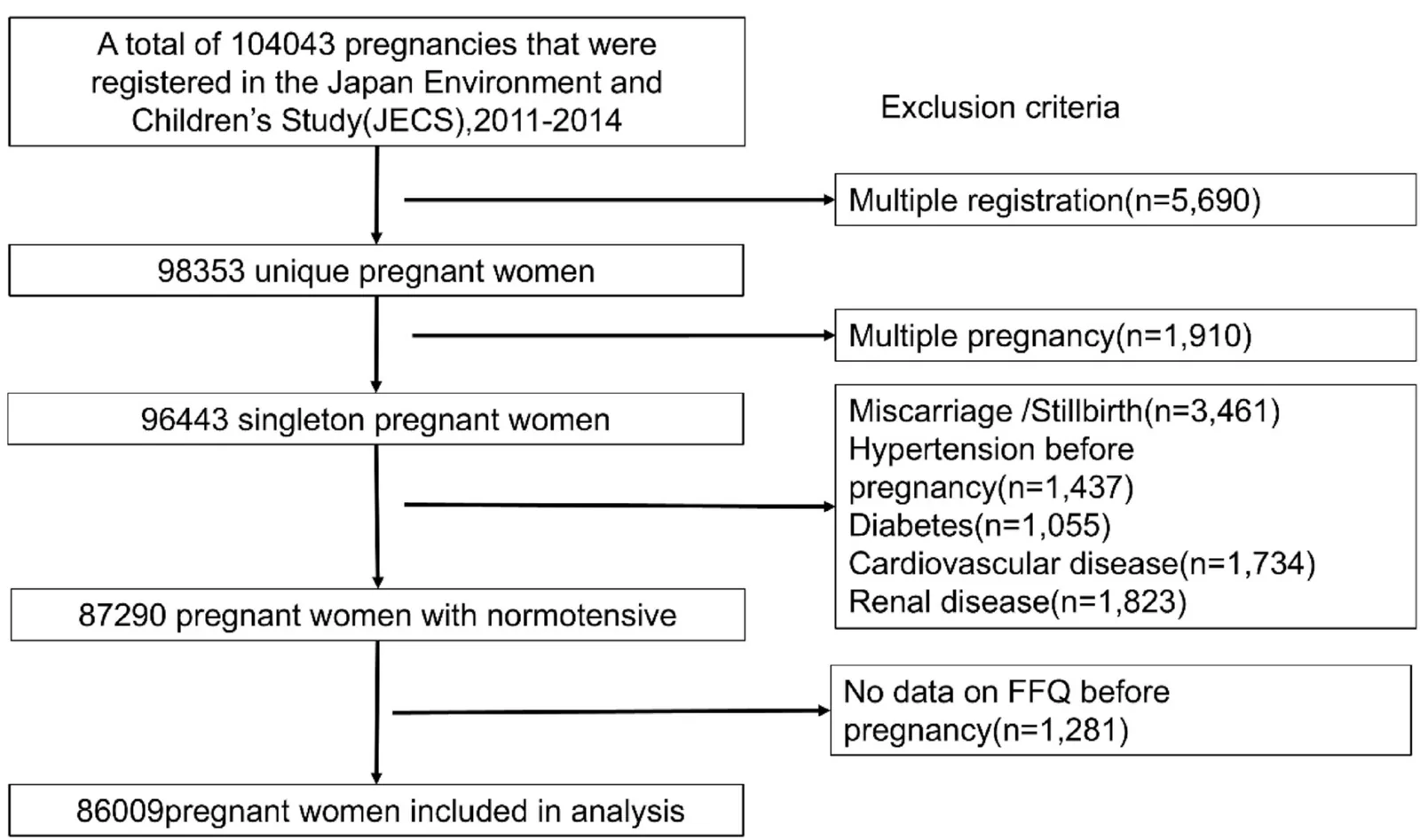
Participant eligibility flowchart. *CVD* cardiovascular disease, *HTN* hypertension, *T2DM* type 2 diabetes

Eligible participants were women registered in the JECS with singleton pregnancies that resulted in delivery. If a woman participated more than once, only the first registration was included, and subsequent records were excluded. The entire dataset comprised 104,043 pregnancy records from 98,353 unique women. After excluding multiple gestations, 96,443 singleton pregnancies remained. Of these, 3,461 pregnancies resulting in miscarriage or stillbirth before 22 weeks of gestation, 1,437 with hypertension before pregnancy, 1,055 with diabetes, 1,734 with heart disease (including congenital heart disease), 1,823 with renal disease and 1,281 with incomplete Food Frequency Questionnaire (FFQ) data were excluded. Consequently, 86,009 maternal records were included in the final analysis (Fig. 1), and analyses of dietary intake during pregnancy included 85,581 participants (Supplementary Fig. 1).

The JECS protocol was reviewed and approved by the Ministry of the Environment’s Institutional Review Board on Epidemiological Studies and the Ethics Committees of all participating institutions. Written informed consent was obtained from all participants.

### Hypertensive disorders of pregnancy

The primary outcome, HDP, was defined according to the diagnostic criteria of the Japan Society for the Study of Hypertension in Pregnancy. HDP was diagnosed when systolic blood pressure was ≥ 140 mmHg or diastolic blood pressure was ≥ 90 mmHg after 20 weeks of gestation in women without a prior history of hypertension [32]. Based on gestational age at onset, HDP was classified as early-onset HDP (Eo-HDP), defined as onset before 32 weeks of gestation, or late-onset HDP (Lo-HDP), defined as onset at or after 32 weeks [33, 34].

Information on HDP was transcribed from medical records via questionnaires completed by physicians, midwives/nurses, and/or Research Co-ordinators. These questionnaires indicated only whether HDP was diagnosed, (“yes” or “no”). Specific HDP phenotypes (i.e., GH, PE, or superimposed PE and eclampsia) were not collected in the JECS.

### Measurements of fish and PUFA intakes

The primary exposure variables in this study were fish and dietary intake of n-3 and n-6 PUFAs before and during pregnancy. In the JECS, these variables were assessed using a self-administered FFQ [35, 36]. The FFQ evaluated dietary intake during the year prior to the baseline survey (early pregnancy; median gestational age, 15.0 weeks [IQR 12.0–19.0]) and during mid to late pregnancy (median gestational age, 27.0 weeks [IQR 25.0–30.0]).

The FFQ recorded the average frequency of food and beverage consumption over the previous year. Nutrient and food group intake (g/day) were estimated using a nutrient calculation software program. Participants reported how often they consumed each food item, with portion sizes categorized as small (50% smaller than standard), medium (standard), or large (50% larger than standard). Consumption frequency was reported using nine categories: less than once per month, 1–3 times per month, 1–2 times per week, 3–4 times per week, 5–6 times per week, every day, 2–3 times per day, 4–6 times per day, and ≥ 7 times per day. Daily fish intake (g/day) was calculated as the product of consumption frequency and standard portion size for each fish item. Daily intake of n–3 and n–6 PUFAs was calculated using the Standard Tables of Fatty Acid Composition in Japan (the JECS dataset does not include information on individual fatty acid subtypes) [37].

Fish and PUFA intake values were log-transformed and energy-adjusted using the residual model [38]. For the 3,342 participants whose fish intake was 0 g/day, we replaced the value with 0.03 g/day, which was one-tenth of the lowest fish intake (0.3 g/day) among the participants (excluding 0 g/day). Similarly, for the 236 participants whose n-3 PUFA intake was 0 g/day, we replaced the value with 0.001 g/day, which was one-tenth of the lowest n-3 PUFA intake (0.01 g/day) among the participants (excluding 0 g/day).

### Statistical analysis

Unless otherwise specified, data are presented as means ± standard deviation or as medians with IQR. Participants were categorized into quintiles based on their fish or PUFA intake. We estimated the risk overall of HDP in relation to fish and PUFA intake and further stratified by Eo- and Lo-HDP groups. Intake was categorized into quintiles, a commonly used approach in studies of PUFAs intake [15, 16], which creates groups with approximately equal numbers of participants. This approach allows the third quintile to represent the group around the median intake and facilitates evaluation of potential non-linear relationships. To evaluate the associations of fish and seafood intake, n-3 and n-6 PUFA intake, and the n-6/n-3 PUFA ratio with HDP risk, logistic regression analysis was performed. Exposure variables were treated as categorical, and multivariable analyses were conducted with adjustment for covariates.

Covariates were selected based on prior literature or theoretical relevance to the outcome [15, 16, 26–28, 39]. The confounders and covariates included the following: maternal age, pre-conception BMI (kg/m²), educational attainment, household income, marital status, alcohol intake, smoking status, physical activity, employment status, intake of eicosapentaenoic acid (EPA) and/or docosahexaenoic acid (DHA) supplements, parity, use of assisted reproductive technology (ART), development of gestational diabetes mellitus (GDM), and history of HDP. To account for overall dietary habits beyond fish intake, we also adjusted for three dietary patterns identified with principal component analysis of food intake data. Maternal age was categorized into four groups: ≤19 years, 20–29 years, 30–39 years, and ≥ 40 years. BMI was classified into three groups: <18.5, 18.5–24.9, and ≥ 25.0 kg/m². Educational level was classified into three groups: 1, junior high or high school; 2, technical junior college, technical/vocational college, or associate degree; 3, bachelor’s degree or postgraduate degree. Household income was categorized into three groups: <4 million, 4–6 million, and ≥ 6 million JPY. Marital status was categorized into two groups: 1, married (including common-law status); 2, single, divorced, or widowed. Alcohol intake was categorized into three groups: 1, never; 2, previously drank but quit; and 3, currently drinking. Smoking status was assessed by self-administered questionnaires and classified as “Never,” “Previously did, but quit before realizing current pregnancy,” “Previously did, but quit after realizing current pregnancy,” and “Currently smoking.” Participants who reported “Currently smoking” were classified as smokers, while all others were classified as nonsmokers. Physical activity was categorized into three groups: low, moderate, and high [40, 41]. Employment status was classified as either yes or no. EPA and/or DHA supplementation was classified as yes for ≥ 1–3 times per week and no for ≤ 2–3 times per month. Parity was classified as nulliparous or multiparous. Pregnancy method was classified as natural conception or ART; ART was defined as in vitro fertilization and/or intracytoplasmic sperm injection or cryopreserved, frozen, or blastocyst embryo transfer. GDM was defined as participants without glucose tolerance disorders in early pregnancy but diagnosed with GDM during the index pregnancy. A history of HDP was defined as the development of HDP in a previous pregnancy but normal blood pressure during early pregnancy of the index pregnancy. The three dietary patterns were derived based on the methodology described in a previous JECS study [42]. The analysis was based on 28 food groups after excluding fish and seaweed. Additionally, women with low or high energy intakes were not excluded from the present study.

For participants with missing covariate data, multiple imputations (MI) were performed to minimize bias [43]. The imputation model included all exposure variables, outcomes, and covariates. Five imputed datasets were created using the R package mice, and results were combined using Rubin’s rules. Statistical significance was defined as a two-sided *p* < 0.05. Adjusted odds ratios (aORs) with 95% confidence intervals (CIs) were calculated.

As a sensitivity analysis to validate the robustness of the main findings, we additionally defined Eo-HDP as onset before 34 weeks of gestation, in accordance with international criteria, and examined associations with fish intake before and during pregnancy [44]. Multicollinearity among covariates was assessed using variance inflation factors (VIFs). All statistical analyses using R version 4.4.3 (Institute for Statistics and Mathematics, Vienna, Austria; www.r-project.org, accessed on 1 Sep 2025).

## Results

### Participant characteristics

Table 1 shows the baseline maternal characteristics according to pre-pregnancy fish intake. The analytic sample included 86,009 women with normal blood pressure in early pregnancy. The mean fish intake was 8.6 g/day in the lowest quintile (Q1) and 52.1 g/day in the highest quintile (Q5). The corresponding mean intake of n-3 PUFAs was 1.1 g/day and 4.7 g/day, respectively. Compared to those in the lowest fish intake group, women in the higher fish intake groups were slightly older (mean difference 1.26 years; 95% CI 1.16–1.37; *p* < 0.001), more likely to be multiparous, had higher educational attainment and household income, were less likely to be current drinkers, and were more often nonsmokers. They were also more likely to have conceived through ART and to be diagnosed with GDM during the index pregnancy. Similar associations were observed for both n–3 and n–6 PUFA intake. Although some other covariates showed statistically significant differences, these were not considered clinically relevant. The baseline characteristics according to parity are presented in Supplementary Table 1. Compared with multiparous women, nulliparous women were slightly younger and were more likely to conceive through ART. Maternal characteristics during pregnancy (*n* = 85,581) are presented in Supplementary Table 2. These characteristics were consistent with those observed before pregnancy. However, overall fish intake during pregnancy was lower than pre-pregnancy levels. For completeness, a comparison of baseline characteristics between included and excluded participants (without multiple imputations) is presented in Supplementary Table 3. Because the excluded group included miscarriage and stillbirth cases, variables collected during mid-to-late pregnancy (e.g., income and education) had higher proportions of missing data in this group.

**Table 1:** Characteristics of women according to pre-pregnancy fish intake (*n* = 86,009)

	Quintile for fish intake				
	1(low)	2	3	4	5(high)
Variable	17,202	17,202	17,202	17,202	17,201
Median intake of nutritons, g/d^a^					
Fish	8.6 (5.2)	20.1 (2.3)	27.5 (2.0)	35.3 (2.6)	52.0 (12.8)
n-3 PUFA	1.1 (1.5)	1.5 (1.0)	2.00 (1.2)	2.6 (1.4)	4.7 (4.1)
n-6 PUFA	12.6 (29.6)	12.6 (16.2)	13.2 (15.1)	14.3 (17.9)	17.3 (18.2)
n-6/n-3 PUFA ratios	14.5 (36.0)	7.7 (4.6)	6.3 (3.9)	5.2 (2.9)	3.8 (2.4)
Age at deliveries, y (SD)	30.2 (5.3)	31.0 (5.0)	31.4 (4.9)	31.6 (4.9)	31.4 (5.0)
Pre-conception BMI: kg/m^2^, n (%)					
<18.5	2875 (16.7)	2751 (16.0)	2786 (16.2)	2771 (16.1)	2864 (16.7)
18.5–25	12,576 (73.1)	12,775 (74.3)	12,690 (73.8)	12,769 (74.2)	12,570 (73.1)
>25	1749 (10.2)	1675 (9.7)	1725 (10.0)	1661 (9.7)	1765 (10.3)
Highest education level, n (%)					
Junior high school or high school	7491 (43.5)	6263 (36.4)	5866 (34.1)	5632 (32.7)	5909 (34.4)
Technical junior college, technical/vocational college, or associate degree	6831 (39.7)	7245 (42.1)	7398 (43.0)	7354 (42.7)	7224 (42.0)
Bachelor’s degree, postgraduate degree	2880 (16.7)	3694 (21.5)	3938 (22.9)	4216 (24.5)	4068 (23.7)
Annual household income (JPY), n (%)					
<4 million	8322 (48.4)	7157 (41.6)	6668 (38.8)	6391 (37.2)	6548 (38.1)
4–6 million	5189 (30.2)	5678 (33.0)	5768 (33.5)	5882 (34.2)	5760 (33.5)
>6 million	3691 (21.5)	4367 (25.4)	4766 (27.7)	4929 (28.7)	4893 (28.4)
Marital status, n (%)					
Married (including common-law marriage)	16,045 (93.3)	16,433 (95.5)	16,492 (95.9)	16,622 (96.6)	16,463 (95.7)
Not married	1157 (6.7)	769 (4.5)	710 (4.1)	580 (3.4)	738 (4.3)
Alcohol intake, n (%)					
Never	6063 (35.2)	5995 (34.9)	5849 (34.0)	5932 (34.5)	5845 (34.0)
Did previously but quit early pregnancy	9541 (55.5)	9286 (54.0)	9419 (54.8)	9502 (55.2)	9876 (57.4)
Current	1598 (9.3)	1921 (11.2)	1934 (11.2)	1768 (10.3)	1480 (8.6)
Currently Smoking during early pregnancy, n (%)	1163 (6.8)	772 (4.5)	693 (4.0)	639 (3.7)	821 (4.8)
Level of physical activity, n (%)					
Low	7526 (43.8)	7280 (42.3)	7363 (42.8)	7352 (42.7)	7468 (43.4)
Moderate	5785 (33.6)	6484 (37.7)	6572 (38.2)	6604 (38.4)	6401 (37.2)
High	3891 (22.6)	3438 (20.0)	3267 (19.0)	3246 (18.9)	3332 (19.4)
Employed, n (%)	9419 (54.8)	9322 (54.2)	9289 (54.0)	9256 (53.8)	9248 (53.8)
Use of EPA and/or DHA supplementation: yes, n (%)	288 (1.7)	295 (1.7)	249 (1.4)	279 (1.6)	309 (1.8)
Parity, n (%)					
Nullipara	8495 (49.4)	7403 (43.0)	7069 (41.1)	7012 (40.8)	7375 (42.9)
Multipara	8707 (50.6)	9799 (57.0)	10,133 (58.9)	10,190 (59.2)	9826 (57.1)
ART, n (%)	385 (2.2)	490 (2.8)	504 (2.9)	568 (3.3)	625 (3.6)
GDM, n (%)	309 (1.8)	338 (2.0)	329 (1.9)	352 (2.0)	401 (2.3)
history of HDP, n (%)	176 (1.0)	211 (1.2)	214 (1.2)	203 (1.1)	167 (1.0)
HDP, n (%)					
Eo-HDP	69 (0.4)	52 (0.3)	54 (0.3)	46 (0.3)	44 (0.3)
Lo-HDP	348 (2.0)	334 (1.9)	323 (1.9)	330 (1.9)	331 (1.9)
^a^Dietary intake before pregnancy. Quintile medians in grams per day adjusted energy intake using the residual method					
*ART* assisted reproductive technology, *BMI* body mass index, *Eo* early-onset, *EPA* eicosapentaenoic, *DHA* docosahexaenoic, *GDM* gestational diabetesmellitus, *HDP* hypertensive disorders of pregnancy, *JPY* Japanese yen, *Lo* late-onset, *PUFA* polyunsaturated fatty acid, *SD* standard deviation					

### Associations between pre-pregnancy fish intake and risk of HDP

**Fig. 2:**
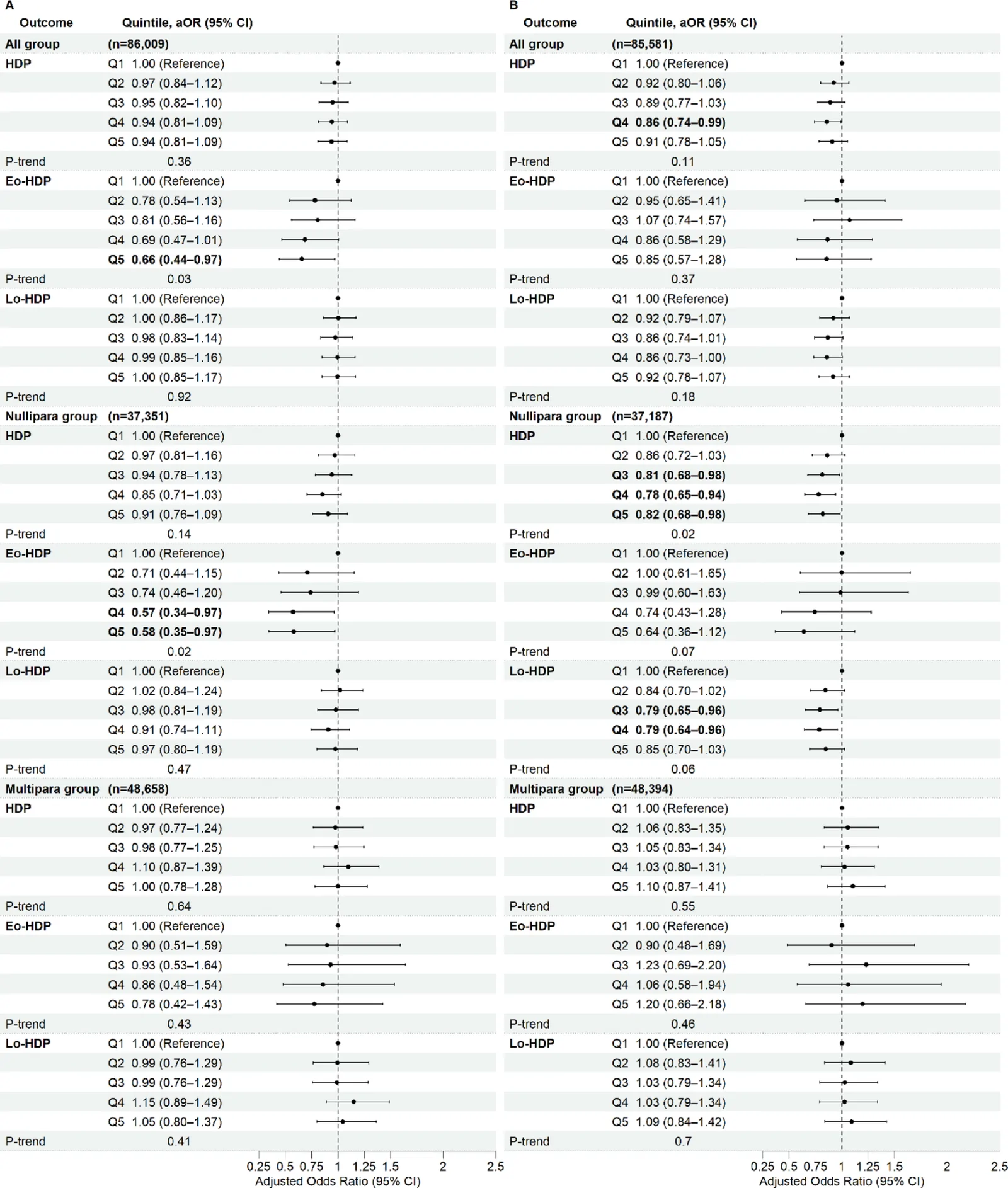
Risk of HDP (aORs and 95% CIs) according to fish intake before and during pregnancy. Adjusted ORs and 95% CIs were estimated using multivariable logistic regression analysis according to quintiles of maternal fish intake, with Q1 as the reference. Panel A presents fish intake before pregnancy (*n* = 86,009), and panel B presents during pregnancy (*n* = 85,581). Results are shown for all HDP, Eo-HDP, and Lo-HDP among all participants and separately among nulliparous and multiparous women. Median fish intake in each quintile is expressed in grams per day after adjustment for total energy intake using the residual method. Bold values indicate statistically significant associations (*P* < 0.05). P for trend indicates the statistical significance of the linear trend across quintiles. Models were adjusted for maternal age, parity, preconception body mass index, highest maternal educational level, annual household income, marital status, alcohol intake, smoking status, physical activity, employment status, use of EPA and/or DHA supplements, history of ART, concurrent GDM, HDP reported at baseline and three dietary patterns. *aOR* adjusted odds ratio, *ART* assisted reproductive technology, *CI* confidence interval, *DHA* docosahexaenoic acid, *Eo-HDP* early-onset hypertensive disorders of pregnancy, *EPA* eicosapentaenoic acid, *GDM* gestational diabetes mellitus, *HDP* hypertensive disorders of pregnancy, *Lo-HDP* late-onset hypertensive disorders of pregnancy

Figure 2A presents the results of multivariable logistic regression analyses of the associations between pre-pregnancy fish intake and HDP risk, with Q1 group as the reference group (*n* = 86,009). No evidence of multicollinearity was observed among the covariates in any of the models (all VIFs < 3). Among all participants, significantly lower aORs for Eo-HDP were observed in the Q5 groups (0.66 [95% CI: 0.44–0.97]). A trend test revealed a significant linear association between fish intake and Eo-HDP (*p* = 0.03). In analyses restricted to nulliparous women, significantly lower aORs for Eo-HDP were also observed in Q4 and Q5 groups (0.57 [95% CI: 0.34–0.97] and 0.58 [95% CI: 0.35–0.97], respectively), with a trend test again revealing a significant linear association (*p* = 0.02). In contrast, no significant reductions in aORs or linear trends were observed among multiparous women.

### Associations between fish intake during pregnancy and risk of HDP

Figure 2B shows the associations between fish intake during pregnancy and the risk of HDP (*n* = 85,581). Among all participants, significantly lower aORs were observed for all HDP in the Q4 group (0.86 [95% CI 0.74–0.99]). Among nulliparous women, significantly lower aORs for all HDP were observed in the Q3, Q4 and Q5 groups, with a trend test indicating a significant linear association. Additionally, lower aORs of Lo-HDP were observed in Q3 and Q4 (0.79 [95% CI 0.65–0.96] and 0.79 [95% CI 0.64–0.96], respectively). In contrast, no significant reductions in aORs or linear associations were observed among multiparous women.

### Associations between PUFA intake before and during pregnancy and risk of HDP

Table 2 summarizes the associations of fish and PUFA intake before and during pregnancy with HDP risk. In the pre-pregnancy analysis, significantly lower aORs for Eo-HDP were observed in the Q5 group of n-3 PUFA intake among all participants (0.63 [95% CI 0.41–0.99]). For n-6 PUFA intake, a significantly higher risk of Eo-HDP was observed in the Q2 group among multiparous women (1.87 [95% CI 1.10–3.19]). Additionally, among all participants, the Q3 group of the n-6/n-3 PUFA ratio was associated with increased risks of both all HDP and Eo-HDP (1.18 [95% CI 1.02–1.36] and 1.55 [95% CI 1.06–2.27], respectively). No other significant associations or linear trends were observed across other quintiles or in parity-stratified analysis. Similarly, in the analyses of PUFA intake during pregnancy, no significant associations or linear trends were observed. Details on the associations for n-3 PUFAs are provided in Supplementary Fig. 2, for n-6 PUFAs in Supplementary Fig. 3, and for the n-6/n-3 PUFA ratios in Supplementary Fig. 4.

**Table 2:** Summary of adjusted ORs for dietary intake of fish and PUFAs before and during pregnancy

		Dietary intake before pregnancy						Dietary intake during pregnancy					
		Quintile for fish or PUFA intake					*P* value for trend	Quintile for fish or PUFA intake					*P* value for trend
		1(low)	2	3	4	5(high)		1(low)	2	3	4	5(high)	
Fish^a^													
All group	HDP	1.00	0.97	0.95	0.94	0.94	0.36	1.00	0.92	0.89	0.86	0.91	0.11
	Eo-HDP	1.00	0.78	0.81	0.69	0.66*	0.03	1.00	0.95	1.07	0.86	0.85	0.37
	Lo-HDP	1.00	1.00	0.98	0.99	1.00	0.92	1.00	0.92	0.86	0.86	0.92	0.18
Nullipara group	HDP	1.00	0.97	0.94	0.85	0.91	0.14	1.00	0.86	0.81*	0.78**	0.82*	0.02
	Eo-HDP	1.00	0.71	0.74	0.57*	0.58*	0.02	1.00	1.00	0.99	0.74	0.64	0.07
	Lo-HDP	1.00	1.02	0.98	0.91	0.97	0.47	1.00	0.84	0.79*	0.79*	0.85	0.06
Multipara group	HDP	1.00	0.97	0.98	1.10	1.00	0.64	1.00	1.06	1.05	1.03	1.10	0.55
	Eo-HDP	1.00	0.90	0.93	0.86	0.78	0.43	1.00	0.90	1.23	1.06	1.20	0.46
	Lo-HDP	1.00	0.99	0.99	1.15	1.05	0.41	1.00	1.08	1.03	1.03	1.09	0.70
n-3 PUFAs^a^													
All group	HDP	1.00	1.04	0.98	1.06	1.01	0.82	1.00	0.89	0.94	1.03	1.01	0.32
	Eo-HDP	1.00	0.93	0.76	0.98	0.63*	0.11	1.00	0.73	0.87	1.07	0.78	0.88
	Lo-HDP	1.00	1.06	1.02	1.07	1.08	0.39	1.00	0.91	0.95	1.02	1.05	0.26
Nullipara group	HDP	1.00	1.03	1.05	1.06	0.96	0.87	1.00	0.89	0.98	1.00	0.94	1.00
	Eo-HDP	1.00	0.89	0.59	1.14	0.68	0.48	1.00	0.85	1.18	1.19	0.86	0.94
	Lo-HDP	1.00	1.06	1.13	1.05	1.01	0.92	1.00	0.89	0.96	0.98	0.95	0.99
Multipara group	HDP	1.00	1.06	0.86	1.05	1.10	0.56	1.00	0.89	0.88	1.06	1.15	0.11
	Eo-HDP	1.00	0.98	0.96	0.79	0.56	0.11	1.00	0.63	0.60	0.98	0.72	0.79
	Lo-HDP	1.00	1.08	0.83	1.10	1.21	0.20	1.00	0.96	0.94	1.08	1.26	0.06
n-6 PUFAs^a^													
All group	HDP	1.00	1.00	1.11	1.07	1.11	0.11	1.00	0.94	0.94	0.93	1.04	0.72
	Eo-HDP	1.00	1.40	0.96	0.97	1.25	0.95	1.00	0.87	0.82	0.91	0.98	0.97
	Lo-HDP	1.00	0.94	1.13	1.09	1.09	0.09	1.00	0.96	0.96	0.94	1.05	0.72
Nullipara group	HDP	1.00	0.89	1.11	1.08	1.08	0.13	1.00	0.93	0.93	0.95	0.95	0.71
	Eo-HDP	1.00	1.04	0.96	1.07	1.19	0.54	1.00	0.64	0.82	0.90	0.90	0.86
	Lo-HDP	1.00	0.87	1.14	1.08	1.07	0.15	1.00	0.98	0.95	0.96	0.96	0.66
Multipara group	HDP	1.00	1.16	1.09	1.06	1.16	0.55	1.00	0.96	0.95	0.90	1.22	0.28
	Eo-HDP	1.00	1.87*	0.95	0.81	1.31	0.53	1.00	1.16	0.83	0.94	1.11	0.94
	Lo-HDP	1.00	1.04	1.11	1.09	1.13	0.37	1.00	0.93	0.97	0.89	1.23	0.23
n-6/n-3 ratio^a^													
All group	HDP	1.00	1.04	1.18*	1.06	1.13	0.11	1.00	1.04	0.98	1.09	1.05	0.41
	Eo-HDP	1.00	1.17	1.55*	1.12	1.30	0.30	1.00	1.19	1.19	1.20	1.20	0.42
	Lo-HDP	1.00	1.02	1.12	1.05	1.11	0.18	1.00	1.02	0.96	1.07	1.03	0.58
Nullipara group	HDP	1.00	1.01	1.17	0.99	1.19	0.11	1.00	0.95	0.92	1.02	1.04	0.46
	Eo-HDP	1.00	1.03	1.40	0.82	1.38	0.42	1.00	0.98	0.89	0.88	1.17	0.67
	Lo-HDP	1.00	1.00	1.14	1.02	1.17	0.15	1.00	0.95	0.93	1.05	1.02	0.53
Multipara group	HDP	1.00	1.08	1.18	1.17	1.02	0.60	1.00	1.19	1.09	1.19	1.03	0.76
	Eo-HDP	1.00	1.36	1.76	1.58	1.08	0.54	1.00	1.50	1.68	1.73	1.15	0.51
	Lo-HDP	1.00	1.04	1.10	1.10	1.01	0.73	1.00	1.14	1.00	1.11	1.01	0.99
Covariates were adjusted for age, previous deliveries, pre-conception BMI, highest maternal educational level, annual household income, marital status, alcohol intake, smoking status, physical activity, employment status, use of EPA and/or DHA, history of ART, concurrent GDM, HDP reported at baseline, and 3 dietary patterns													
^a^Quintile medians in grams per day adjusted energy intake using the residual method													
*Eo* early-onset, *HDP* hypertensive disorders of pregnancy, *Lo* late-onset. *p < 0.05, **p < 0.01, *** < *p* < 0.001													

### Analysis using < 34 weeks of gestation as the cutoff for Eo-HDP

Supplementary Fig. 5A presents the associations between pre-pregnancy fish intake and the risk of HDP when Eo-HDP was defined as onset before 34 weeks of gestation. Among all participants, a significantly lower aOR for Eo-HDP was observed in the Q4 group (0.72 [95% CI 0.52–0.99]). Supplementary Fig. 5B shows the associations between fish intake during pregnancy and HDP risk using the same < 34-week definition. Among all participants, a significant reduction in the risk of all HDP was observed in the Q4 group. Among nulliparous women, significantly lower risks of all HDP were observed in the Q3, Q4, and Q5 groups, with a significant linear trend. For Eo-HDP, a significant linear association was detected. Additionally, significantly lower risks of Eo-HDP and Lo-HDP were observed in Q5 (0.59 [95% CI 0.37–0.92]) and Q4 (0.80 [95% CI 0.65–0.98]), respectively.

## Discussion

In this study, we examined the associations between fish and PUFAs intake and the risk of HDP. Fish intake both before and during pregnancy was inversely associated with HDP risk among 86,009 participants in the JECS. Notably, the greatest risk reduction was observed for Eo-HDP in nulliparous women in the Q4 group of pre-pregnancy fish intake. During pregnancy, the largest risk reduction occurred for Lo-HDP in nulliparous women in Q4.

Our findings are consistent with previous epidemiological studies, indicating that higher fish intake may lower the risk of HDP. A large Norwegian prospective cohort study also reported that a seafood diet during pregnancy was associated with a lower risk of HDP [19]. In contrast, a Danish nested case–control study found no significant association between fish intake and HDP [18]. Although epidemiological findings regarding fish intake have been mixed, previous studies have more consistently suggested protective effects of n-3 PUFA intake against PE. A case–control study in China reported that higher EPA and DHA intake during pregnancy was associated with a reduced risk of PE [17]. In the Danish National Birth Cohort, higher DHA intake in early pregnancy and adherence to the recommended EPA and DHA intake were linked to a lower risk of PE, particularly severe PE [16]. Similarly, the U.S. Nurses’ Health Study II showed that higher pre-pregnancy EPA + DHA intake was associated with a lower risk of PE [15]. Collectively, these findings support the protective effects of fish and n-3 PUFA intake against HDP.

HDP is thought to arise from immunogenic maladaptation at the maternal–fetal interface. In HDP, insufficient cytotrophoblast invasion and inadequate replacement of vascular smooth muscle and endothelial cells lead to impaired spiral artery remodeling, resulting in increased vascular resistance, decreased uteroplacental blood flow, and hypoxia [45, 46]. Under hypoxic conditions, trophoblasts increase production of soluble Fms-like tyrosine kinase-1 (sFlt-1), and decrease placental growth factor (PlGF), triggering antiangiogenic effects that worse placental hypoxia and further upregulate sFlt-1 and suppress PlGF levels [47]. Circulating sFlt-1 induces maternal endothelial dysfunction, resulting in hypertension, increased coagulation, vascular permeability, and the clinical features of HDP.

Administration of LDA before 16 weeks of gestation has been reported to reduce the incidence of HDP [20, 21]. Although recommendations vary slightly, major guidelines all recommend LDA use for women at high risk of developing PE or HDP [48–50]. The protective effect of LDA is believed to arise from inhibition of platelet aggregation and suppression of prostacyclin I_2_ production. Similarly, n-3 PUFAs have been reported to exert anticoagulant effects. Increased intake of n-3 PUFAs, including EPA and DHA, results in their dose-dependent incorporation into inflammatory cell phospholipids, partly displacing arachidonic acid, which may contribute to enhanced vasodilation and antithrombotic activity [22, 23]. Based on our findings, we hypothesize that increased fish intake enhances the metabolism of n-3 PUFAs, including EPA and DHA, slightly shifting the hemostatic balance toward fibrinolysis. This mechanism, akin to that of LDA, may explain the protective association between fish intake and the reduced risk of HDP.

In this study, pre-pregnancy fish intake was associated with a reduced risk of Eo-HDP, whereas no significant reduction was observed for Lo-HDP. Two explanations may account for this difference. First, Eo-HDP is characterized by impaired spiral artery remodeling, whereas Lo-HDP reflects oxidative stress–related placental dysfunction associated with maternal metabolic abnormalities [51–53]. Second, it is possible that fish intake reduced the risk of both Eo-HDP and Lo-HDP, but this effect may have shifted the onset of Eo-HDP to later gestational weeks. Such a shift could result in cases initially classified as Eo-HDP being reclassified as Lo-HDP, potentially offsetting the apparent risk reduction for Lo-HDP. A similar phenomenon has been reported with LDA use [21]. In contrast to pre-pregnancy intake, fish intake during pregnancy was associated with a lower risk of Lo-HDP but not Eo-HDP. This may reflect differences in the timing of exposure. Pre-pregnancy fish intake may influence the pathogenesis of Eo-HDP, whereas fish intake during pregnancy may be more relevant to the development of Lo-HDP. Given that maternal obesity and related metabolic factors are major risk factors for Lo-HDP, the influence of fish intake may be relatively modest. The inverse association observed between pre-pregnancy fish intake and Eo-HDP was stronger in magnitude than the association between fish intake during pregnancy and Lo-HDP although both were statistically significant. It is also well-known that pregnant women tend to avoid fish consumption during pregnancy, a trend observed in our cohort. Notably, women in the lowest fish intake group—those who later developed Eo-HDP—may have slightly increased their fish intake following the start of prenatal visits and dietary counseling at medical institutions. However, the overall decline in fish intake during pregnancy may explain the lack of a significant association between fish intake during pregnancy and Eo-HDP. Therefore, reduced fish consumption during pregnancy is unlikely to lower the risk of HDP. Overall, these findings suggest that fish intake before and during pregnancy may reduce the risk of Eo-HDP and Lo-HDP, respectively. Maintaining a consistent level of fish consumption throughout both periods may be important HDP prevention.

Most of previous studies have reported that intake of EPA and DHA may contribute to the prevention of HDP [15–17]. In contrast, the present study found significant associations between total n-3 PUFA intake and HDP in certain models or subgroups. This discrepancy may be due to differences in the types and precision of fatty acid classifications evaluated. The n-3 PUFAs include several components such as α-linolenic acid, EPA, and DHA, each exhibiting distinct physiological functions depending on chain length and degree of unsaturation. Among these, EPA and DHA possess particularly strong anti-inflammatory and vasodilatory properties [23]. In the JECS, individual fatty acid intakes were not measured directly; thus, the analysis relied on total n-3 PUFA intake, which also included components with relatively low biological activity. Consequently, the potential risk-reducing effect may have been diluted. Additionally, n-3 PUFA intake was estimated based on food composition tables, which may not accurately capture interindividual variation in actual intake levels.

In stratified analyses, a greater number of significant associations were observed among nulliparous women. This may reflect the fact that HDP occurs most frequently in nulliparous women, and nulliparity itself being major risk factors. Indeed, PE risk is higher in nulliparous women [54], while multiparous women with a history of HDP, especially PE, have a markedly increased risk of recurrence [55]. Moreover, PE recurs in 13.1% of women after an initial episode [56], with an increased risk in later pregnancies among those previously affected by PE, GH, or HELLP syndrome [57]. Given these observations, the limited significant risk reduction observed among multiparous women in this study is likely due to heterogeneity in their baseline risk profiles. The multiparous group likely included both women at high risk due to prior HDP and those at low risk without such history, which may have attenuated the statistical detectability of the effects of fish and PUFA intake.

Women with a history of HDP and those with pre-existing hypertension were excluded from the present study. Accordingly, the HDP cases included in this study largely correspond to PE as defined in previous studies [11, 13–17, 19]. To align the terminology with previous JECS studies [26–28], we used the term “HDP” rather than “PE” throughout this manuscript. In this study, Eo-HDP was defined as onset before 32 weeks of gestation. However, the International Society of Hypertension in Pregnancy uses a 34-week cutoff to distinguish between Eo- and Lo-HDP, based on a 2012 international survey [44]. Despite this, HDP cases occurring before 32 weeks of gestation are known to present particular challenges in terms of prognosis and complications. Brodowski et al. developed a consensus on clinical severity assessment specifically for PE developing before 32 weeks, finding that the conventional 34-week criterion did not adequately identify this distinct subgroup [33]. Similarly, Suzuki et al. established gestational age–specific reference values for serum sFlt-1/PlGF ratios in Japanese pregnant women and reported that PE cases with onset before 32 weeks had a significantly higher frequency of abnormal values than those with later onset [34]. Although only a few studies have employed the 32-week cutoff, this approach offers valuable insights for refining management criteria to better reflect clinical realities. In this study, focusing on HDP pathogenesis originating from impaired spiral artery remodeling, defining Eo-HDP as onset before 32 weeks may have allowed a more accurate distinction between Eo and Lo forms of HDP, which likely involve different underlying mechanisms. Therefore, the antithrombotic effects of n-3 PUFAs derived from fish intake may have contributed to the enhanced risk reduction observed for Eo-HDP. Notably, sensitivity analyses using the 34-week cutoff demonstrated consistent trends—a reduced risk of Eo-HDP associated with pre-pregnancy fish intake and a reduced risk of Lo-HDP associated with fish intake during pregnancy—supporting the robustness of this hypothesis.

The primary strength of this study lies in its large sample size. To our knowledge, this is the largest study to date examining the association between dietary intake of fish and n-3 PUFAs and the risk of HDP. Moreover, this is the first study to apply MI to handle missing data across multiple variables, thereby minimizing bias and enhancing the validity and precision of the results. Notably, in the 2025 Dietary Guidelines Advisory Committee’s systematic review conducted with support from the USDA’s Nutrition Evidence Systematic Review team, no observational studies were identified as having a low risk of bias regarding the handling of missing data; most lacked sufficient descriptions of missing data procedures [58]. The consistency of our findings with previous reports, even after applying MI, supports the robustness and reproducibility of the results.

This study has several limitations. First, the JECS FFQ did not allow detailed identification of fish species other than marine fish. This likely reflects the traditional Japanese dietary pattern, in which the fish most consumed are predominantly marine fish [59]. Consequently, PUFA intake derived from fish other than marine fish could not be estimated separately. Second, intake of n-3 PUFAs was estimated as a total value because data on specific components, such as EPA and DHA, were not available. Likewise, n-6PUFAs intake was assessed only as a total value. Therefore, the individual effects of each fatty acid fraction could not be clearly evaluated. Third, the study did not assess individual HDP subtypes, such as GH, PE, and HELLP syndrome, limiting the ability to analyze potential pathophysiological differences among these subtypes. Fourth, in the stratified analyses by gestational age at onset, Eo-HDP was defined using a 32-week cutoff instead of the internationally common 34-week threshold, which may limit direct comparability with other studies. Fifth, we did not perform restricted cubic spline analyses, precluding the evaluation of potential non-linear associations between fish or PUFA intake and HDP, as well as the identification of the optimal intake levels. Finally, given the multiple comparisons, some of the observed differences may be attributable to chance.

In conclusion, this study demonstrated that fish intake before and during pregnancy was associated with a reduced risk of HDP. Notably, pre-pregnancy fish intake was significantly associated with a lower risk of Eo-HDP among nulliparous women, suggesting that the anti-inflammatory and antithrombotic effects of n-3 PUFAs, similar to those of LDA, may be involved in the underlying mechanisms. Conversely, fish intake during pregnancy was linked to a reduced risk of Lo-HDP. These findings underscore the importance of maintaining consistent fish consumption from preconception through pregnancy for the prevention of HDP. Furthermore, our findings provide valuable evidence to inform global nutritional guidance, including preconception care. In Japan, where fish consumption has traditionally been high but has shown a recent declining trend, these results offer practical and realistic insights for developing dietary counseling strategies aimed at maternal health.

## Supplementary Information

Below is the link to the electronic supplementary material.

Supplementary Material 1.

## Data Availability

Data are unsuitable for public deposition due to ethical restrictions and legal framework of Japan. It is prohibited by the Act on the Protection of Personal Information (Act No. 57 of 30 May 2003, amendment on 9 September 2015) to publicly deposit the data containing personal information. Ethical Guidelines for Medical and Health Research Involving Human Subjects enforced by the Japan Ministry of Education, Culture, Sports, Science and Technology and the Ministry of Health, Labour and Welfare also restricts the open sharing of the epidemiologic data. All inquiries about access to data should be sent to: jecs-en@nies.go.jp. The person responsible for handling enquiries sent to this e-mail address is Dr Shoji F. Nakayama, JECS Programme Office, National Institute for Environmental Studies. In addition, the data analysis performed for this manuscript was approved by the Ethics Committee of Gunma University too.

## Abbreviations

Eo-HDP: Early-Onset Hypertensive Disorders of Pregnancy
LDA: Low-Dose Aspirin
Lo-HDP: Late-Onset Hypertensive Disorders of Pregnancy
MI: Multiple Imputation
n-3 PUFA: Omega-3 Polyunsaturated Fatty Acids
n-6 PUFA: Omega-6 Polyunsaturated Fatty Acids
PlGF: Placental Growth Factor
sFlt-1: Soluble Fms-Like Tyrosine Kinase-1
ISSHP: International Society for the Study of Hypertension in Pregnancy
