# Supplementary material for: Association of the maternal fish and PUFA intake with the risk of hypertensive disorders of pregnancy: a nationwide birth cohort—The Japan Environment and Children’s Study (JECS)
Source: Eur J Nutr. 2026 Sep 8;65(7):249. doi: 10.1007/s00394-026-04103-7 (PMC13553602; doi:10.1007/s00394-026-04103-7)
Supplement: Supplementary file 1 [file 394_2026_4103_MOESM1_ESM.docx]

Association of the Maternal Fish and PUFA Intake With the Risk of Hypertensive Disorders of Pregnancy: A Nationwide Birth Cohort—The Japan Environment and Children’s Study (JECS)
Takuya Inouye

Supplementary Material

Figure legend

Supplementary Figure.1 Participant eligibility flowchart for the FFQ during pregnancy

Abbreviation: CVD, cardiovascular disease; HTN, hypertension; T2DM, type 2 diabetes.

.

| **Supplementary Table 1.** Baseline characteristics of nulliparous and multiparous women (n=86,009) | | |
| --- | --- | --- |
|  | nulliparous | multipara |
| Variable | 37351 | 48658 |
| Median intake of nutritons, g/d^1^ |  |  |
| Fish | 28.1 (16.7) | 29.2 (15.4) |
| n-3 PUFA | 2.6 (2.5) | 2.4 (2.5) |
| n-6PUFA | 14.6 (22.9) | 13.6 (17.8) |
| n-6/n-3 PUFA ratios | 7.8 (15.8) | 7.3 (17.6) |
| Age at deliveries, y (SD) | 29.7 (5.2) | 32.2 (4.6) |
| Pre-conception BMI: kg/m^2^, n (%) |  |  |
| <18.5 | 6621 (17.7) | 7429 (15.3) |
| 18.5-25 | 27590 (73.9) | 35792 (73.6) |
| >25 | 3140 (8.4) | 5437 (11.2) |
| Highest education level, n (%) |  |  |
| Junior high school or high school | 12441 (33.3) | 18720 (38.5) |
| Technical junior college, technical/vocational college, or associate degree | 15642 (41.9) | 20409 (41.9) |
| Bachelor's degree, postgraduate degree | 9268 (24.8) | 9529 (19.6) |
| Annual household income (JPY), n (%) |  |  |
| <4 million | 15260 (40.9) | 19828 (40.7) |
| 4-6 million | 11756 (31.5) | 16520 (34.0) |
| >6 million | 10335 (27.7) | 12310 (25.3) |
| Marital status, n (%) |  |  |
| Married (including common-law marriage) | 34236 (91.7) | 47820 (98.3) |
| Not married | 3115 (8.3) | 838 (1.7) |
| Alcohol intake, n (%) |  |  |
| Never | 12078 (32.3) | 17606 (36.2) |
| Did previously but quit early pregnancy | 22328 (59.8) | 25296 (52.0) |
| Current | 2945 (7.9) | 5756 (11.8) |
|  |  |  |
| Currently Smoking during early pregnancy, n (%) | 1384 (3.7) | 2706 (5.6) |
| Level of physical activity, n (%) |  |  |
| Low | 18089 (48.4) | 18900 (38.8) |
| Moderate | 10262 (27.5) | 21584 (44.4) |
| High | 9000 (24.1) | 8174 (16.8) |
|  |  |  |
| Employed, n (%) | 22925 (61.4) | 23611 (48.5) |
| Use of EPA and/or DHA supplementation: yes, n (%) | 832 (2.2) | 589 (1.2) |
|  |  |  |
| ART, n (%) | 1776 (4.8) | 796 (1.6) |
| GDM, n (%) | 723 (1.9) | 1005 (2.1) |
| history of HDP, n (%) | 0 (0.0) | 970 (2.0) |
| HDP, n (%) |  |  |
| Eo-HDP, n (%) | 148 (0.4) | 118 (0.2) |
| Lo-HDP, n (%) | 1026 (2.7) | 639 (1.3) |
| ^1^Dietary intake before pregnancy. Quintile medians in grams per day adjusted energy intake using the residual method. | | |
| ART, assisted reproductive technology; BMI, body mass index; Eo, early-onset; EPA, eicosapentaenoic; DHA, docosahexaenoic; GDM, gestational diabetes mellitus; HDP, hypertensive disorders of pregnancy; JPY, Japanese yen; Lo, late-onset; PUFA, polyunsaturated fatty acid; SD, standard deviation. | | |

| **Supplementary Table 2.** Characteristics of women according to fish intake during pregnancy,  with early-onset HDP defined as occurring before 32 weeks of gestation (n=85,581) | | | | | |
| --- | --- | --- | --- | --- | --- |
|  | Quintile for fish intake | | | | |
|  | 1(low) | 2 | 3 | 4 | 5(high) |
| Variable | 17117 | 17116 | 17116 | 17116 | 17116 |
| Median intake of nutritons, g/d^1^ |  |  |  |  |  |
| Fish | 6.3 (4.2) | 15.8 (1.8) | 21.5 (1.6) | 27.4 (1.9) | 39.2 (9.1) |
| n-3 PUFA | 1.0 (1.3) | 1.3 (1.0) | 1.7 (1.1) | 2.3 (1.2) | 4.3 (3.7) |
| n-6PUFA | 11.4 (24.7) | 11.5 (14.6) | 12.2 (14.4) | 13.1 (13.2) | 17.1 (20.1) |
| n-6/n-3 PUFA ratios | 13.6 (26.7) | 8.0 (5.3) | 6.5 (3.5) | 5.3 (2.8) | 4.0 (2.5) |
| Age at deliveries, y (SD) | 30.3 (5.2) | 31.1 (5.0) | 31.3 (5.0) | 31.6 (4.9) | 31.4 (5.1) |
| Pre-conception BMI: kg/m^2^, n (%) |  |  |  |  |  |
| <18.5 | 2849 (16.6) | 2771 (16.2) | 2743 (16.0) | 2782 (16.3) | 2858 (16.7) |
| 18.5-25 | 12471 (72.9) | 12724 (74.3) | 12775 (74.6) | 12630 (73.8) | 12467 (72.8) |
| >25 | 1797 (10.5) | 1621 (9.5) | 1598 (9.3) | 1704 (10.0) | 1791 (10.5) |
| Highest education level, n (%) |  |  |  |  |  |
| Junior high school or high school | 7388 (43.2) | 6160 (36.0) | 5793 (33.8) | 5576 (32.6) | 6065 (35.4) |
| Technical junior college, technical/vocational college, or associate degree | 6875 (40.2) | 7339 (42.9) | 7325 (42.8) | 7282 (42.5) | 7062 (41.3) |
| Bachelor's degree, postgraduate degree | 2854 (16.7) | 3617 (21.1) | 3998 (23.4) | 4258 (24.9) | 3989 (23.3) |
| Annual household income (JPY), n (%) |  |  |  |  |  |
| <4 million | 8218 (48.0) | 7016 (41.0) | 6760 (39.5) | 6379 (37.3) | 6521 (38.1) |
| 4-6 million | 5206 (30.4) | 5661 (33.1) | 5706 (33.3) | 5755 (33.6) | 5806 (33.9) |
| >6 million | 3693 (21.6) | 4439 (25.9) | 4650 (27.2) | 4982 (29.1) | 4789 (28.0) |
| Marital status, n (%) |  |  |  |  |  |
| Married (including common-law marriage) | 16013 (93.6) | 16351 (95.5) | 16449 (96.1) | 16497 (96.4) | 16362 (95.6) |
| Not married | 1104 (6.4) | 765 (4.5) | 667 (3.9) | 619 (3.6) | 754 (4.4) |
| Alcohol intake, n (%) |  |  |  |  |  |
| Never | 5826 (34.0) | 5613 (32.8) | 5728 (33.5) | 5595 (32.7) | 5813 (34.0) |
| Did previously but quit early pregnancy | 10871 (63.5) | 10991 (64.2) | 10877 (63.5) | 11035 (64.5) | 10864 (63.5) |
| Current | 420 (2.5) | 512 (3.0) | 511 (3.0) | 486 (2.8) | 439 (2.6) |
|  |  |  |  |  |  |
| Currently Smoking during early pregnancy, n (%) | 1101 (6.4) | 795 (4.6) | 732 (4.3) | 628 (3.7) | 767 (4.5) |
| Level of physical activity, n (%) |  |  |  |  |  |
| Low | 7484 (43.7) | 7230 (42.2) | 7271 (42.5) | 7365 (43.0) | 7384 (43.1) |
| Moderate | 5894 (34.4) | 6414 (37.5) | 6514 (38.1) | 6521 (38.1) | 6427 (37.6) |
| High | 3739 (21.8) | 3472 (20.3) | 3331 (19.5) | 3230 (18.9) | 3305 (19.3) |
|  |  |  |  |  |  |
| Employed, n (%) | 9630 (56.3) | 9324 (54.5) | 9249 (54.0) | 9132 (53.4) | 8997 (52.6) |
| Use of EPA and/or DHA supplementation: yes, n (%) | 281 (1.6) | 276 (1.6) | 272 (1.6) | 258 (1.5) | 323 (1.9) |
| Parity, n (%) |  |  |  |  |  |
| Nullipara | 8257 (48.2) | 7383 (43.1) | 7146 (41.7) | 7073 (41.3) | 7328 (42.8) |
| Multipara | 8860 (51.8) | 9733 (56.9) | 9970 (58.3) | 10043 (58.7) | 9788 (57.2) |
| ART, n (%) | 385 (2.2) | 479 (2.8) | 502 (2.9) | 575 (3.4) | 612 (3.6) |
| GDM, n (%) | 305 (1.8) | 301 (1.8) | 329 (1.9) | 376 (2.2) | 422 (2.5) |
| history of HDP, n (%) | 176 (1.0) | 193 (1.1) | 203 (1.2) | 197 (1.2) | 199 (1.2) |
| HDP, n (%) |  |  |  |  |  |
| Eo-HDP, n (%) | 55 (0.3) | 50 (0.3) | 57 (0.3) | 47 (0.3) | 47 (0.3) |
| Lo-HDP, n (%) | 373 (2.2) | 327 (1.9) | 309 (1.8) | 311 (1.8) | 337 (2.0) |
| ^1^Dietary intake before pregnancy. Quintile medians in grams per day adjusted energy intake using the residual method. | | | | | |
| ART, assisted reproductive technology; BMI, body mass index; Eo, early-onset; EPA, eicosapentaenoic; DHA, docosahexaenoic; GDM, gestational diabetes mellitus; HDP, hypertensive disorders of pregnancy; JPY, Japanese yen; Lo, late-onset; PUFA, polyunsaturated fatty acid; SD, standard deviation. | | | | | |

| **Supplementary table 3.** Comparison of excluded participants without multiple imputation (n=98,353) | | |
| --- | --- | --- |
|  | inclusion | exclusion |
| Variable | 86009 | 12344 |
| Age at deliveries, y(SD) | 31.12 (5.05) | 31.87 (5.32) |
| missing n (%) | 2(0.0) | 1974 (16.0) |
| Pre-conception BMI: kg/m2, n (%) |  |  |
| <18.5 | 14044 (16.3) | 1860 (15.1) |
| 18.5-25 | 63357 (73.7) | 7993 (64.8) |
| >25 | 8571 (10.0) | 1866 (15.1) |
| missing n (%) | 37 (0.0) | 625 (5.1) |
| Highest education level, n (%) |  |  |
| Junior high school or high school | 30563 (35.5) | 3026 (24.5) |
| Technical junior college, technical/vocational college, or associate degree | 35518 (41.3) | 3594 (29.1) |
| Bachelor's degree, postgraduate degree | 18548 (21.6) | 1694 (13.7) |
| missing | 1380 (1.6) | 4030 (32.6) |
| Annual household income (JPY), n (%) |  |  |
| <4 million | 31638 (36.8) | 3084 (25.0) |
| 4-6 million | 26130 (30.4) | 2503 (20.3) |
| >6 million | 21194 (24.6) | 2165 (17.5) |
| missing | 7047 (8.2) | 4592 (37.2) |
| Marital status, n (%) |  |  |
| Married (including common-law marriage) | 81715 (95.0) | 8683 (70.3) |
| Not married | 3912 (4.5) | 442 (3.6) |
| missing | 382 (0.4) | 3219 (26.1) |
| Alcohol intake, n (%) |  |  |
| Never | 29588 (34.4) | 3037 (24.6) |
| Did previously but quit early pregnancy | 47476 (55.2) | 5145 (41.7) |
| Current | 8675 (10.1) | 929 (7.5) |
| missing | 270 (0.3) | 3233 (26.2) |
|  |  |  |
| Currently Smoking during early pregnancy, n (%) | 4047 (4.7) | 484 (3.9) |
| missing | 641 (0.7) | 3252 (26.3) |
| Level of physical activity, n (%) |  |  |
| Low | 28760 (33.4) | 3042 (24.6) |
| Moderate | 24944 (29.0) | 2556 (20.7) |
| High | 13353 (15.5) | 1550 (12.6) |
| missing | 18952 (22.0) | 5196 (42.1) |
|  |  |  |
| Employed, n (%) | 45669 (53.1) | 4232 (34.3) |
| missing | 1653 (1.9) | 4069 (33.0) |
|  |  |  |
| Use of EPA and/or DHA supplementation: yes, n (%) | 1390 (1.6) | 154 (1.2) |
| missing | 1793 (2.1) | 4081 (33.1) |
| Parity, n (%) |  |  |
| Nullipara | 36953 (43.0) | 5300 (42.9) |
| Multipara | 48286 (56.1) | 6327 (51.3) |
| missing | 770 (0.9) | 717 (5.8) |
|  |  |  |
| ART, n (%) | 2562 (3.0) | 571 (4.6) |
| missing | 366 (0.4) | 671 (5.4) |
|  |  |  |
| GDM, n (%) | 1729 (2.0) | 874 (7.1) |
| missing | 0 (0.0) | 2208 (17.9) |
|  |  |  |
| history of HDP, n (%) | 989 (1.1) | 332 (2.7) |
| missing | 0 (0.0) | 3170 (25.7) |
| ART, assisted reproductive technology; BMI, body mass index; EPA, eicosapentaenoic; DHA, docosahexaenoic; GDM, gestational diabetes mellitus; HDP, hypertensive disorders of pregnancy; JPY, Japanese yen; SD, standard deviation. | | |

Figure legend

Supplementary Figure 2 Risk of HDP (aORs and 95% CIs) according to n-3 PUFA intake before and during pregnancy

Adjusted ORs and 95% CIs were estimated using multivariable logistic regression analysis according to quintiles of maternal fish intake, with Q1 as the reference. Panel A presents n-3 PUFA intake before pregnancy (n= 86,009), and panel B presents during pregnancy (n= 85,581). Results are shown for all HDP, Eo-HDP, and Lo-HDP among all participants and separately among nulliparous and multiparous women. Median n-3 PUFA intake in each quintile is expressed in grams per day after adjustment for total energy intake using the residual method. Bold values indicate statistically significant associations (P < 0.05). P for trend indicates the statistical significance of the linear trend across quintiles. Models were adjusted for maternal age, parity, preconception body mass index, highest maternal educational level, annual household income, marital status, alcohol intake, smoking status, physical activity, employment status, use of EPA and/or DHA supplements, history of ART, concurrent GDM, HDP reported at baseline and three dietary patterns.

Abbreviations: aOR, adjusted odds ratio; ART, assisted reproductive technology; CI, confidence interval; DHA, docosahexaenoic acid; Eo-HDP, early-onset hypertensive disorders of pregnancy; EPA, eicosapentaenoic acid; GDM, gestational diabetes mellitus; HDP, hypertensive disorders of pregnancy; Lo-HDP, late-onset hypertensive disorders of pregnancy.

Figure legend

Supplementary Figure 3 Risk of HDP (aORs and 95% CIs) according to n-6 PUFA intake before and during pregnancy

Adjusted ORs and 95% CIs were estimated using multivariable logistic regression analysis according to quintiles of maternal fish intake, with Q1 as the reference. Panel A presents n-6 PUFA intake before pregnancy (n= 86,009), and panel B presents during pregnancy (n= 85,581). Results are shown for all HDP, Eo-HDP, and Lo-HDP among all participants and separately among nulliparous and multiparous women. Median n-6 PUFA intake in each quintile is expressed in grams per day after adjustment for total energy intake using the residual method. Bold values indicate statistically significant associations (P < 0.05). P for trend indicates the statistical significance of the linear trend across quintiles. Models were adjusted for maternal age, parity, preconception body mass index, highest maternal educational level, annual household income, marital status, alcohol intake, smoking status, physical activity, employment status, use of EPA and/or DHA supplements, history of ART, concurrent GDM, HDP reported at baseline and three dietary patterns.

Abbreviations: aOR, adjusted odds ratio; ART, assisted reproductive technology; CI, confidence interval; DHA, docosahexaenoic acid; Eo-HDP, early-onset hypertensive disorders of pregnancy; EPA, eicosapentaenoic acid; GDM, gestational diabetes mellitus; HDP, hypertensive disorders of pregnancy; Lo-HDP, late-onset hypertensive disorders of pregnancy.

Figure legend

Supplementary Figure 4 Risk of HDP (aORs and 95% CIs) according to n-6/n-3 PUFA ratios before and during pregnancy

Adjusted ORs and 95% CIs were estimated using multivariable logistic regression analysis according to quintiles of maternal fish intake, with Q1 as the reference. Panel A presents n-6/n-3 PUFA ratios before pregnancy (n= 86,009), and panel B presents during pregnancy (n= 85,581). Results are shown for all HDP, Eo-HDP, and Lo-HDP among all participants and separately among nulliparous and multiparous women. Median n-6/n-3 PUFA ratios in each quintile is expressed in grams per day after adjustment for total energy intake using the residual method. Bold values indicate statistically significant associations (P < 0.05). P for trend indicates the statistical significance of the linear trend across quintiles. Models were adjusted for maternal age, parity, preconception body mass index, highest maternal educational level, annual household income, marital status, alcohol intake, smoking status, physical activity, employment status, use of EPA and/or DHA supplements, history of ART, concurrent GDM, HDP reported at baseline and three dietary patterns.

Abbreviations: aOR, adjusted odds ratio; ART, assisted reproductive technology; CI, confidence interval; DHA, docosahexaenoic acid; Eo-HDP, early-onset hypertensive disorders of pregnancy; EPA, eicosapentaenoic acid; GDM, gestational diabetes mellitus; HDP, hypertensive disorders of pregnancy; Lo-HDP, late-onset hypertensive disorders of pregnancy.

Supplementary Figure 5 Risk of HDP (before 34weeks was defined as early-onset) according to fish intake before and during pregnancy

Adjusted ORs and 95% CIs were estimated using multivariable logistic regression analysis according to quintiles of maternal fish intake, with Q1 as the reference. Panel A presents fish intake before pregnancy (n= 86,009), and panel B presents during pregnancy (n= 85,581). Results are shown for all HDP, Eo-HDP, and Lo-HDP among all participants and separately among nulliparous and multiparous women. Median fish intake in each quintile is expressed in grams per day after adjustment for total energy intake using the residual method. Bold values indicate statistically significant associations (P < 0.05). P for trend indicates the statistical significance of the linear trend across quintiles. Models were adjusted for maternal age, parity, preconception body mass index, highest maternal educational level, annual household income, marital status, alcohol intake, smoking status, physical activity, employment status, use of EPA and/or DHA supplements, history of ART, concurrent GDM, HDP reported at baseline and three dietary patterns.

Abbreviations: aOR, adjusted odds ratio; ART, assisted reproductive technology; CI, confidence interval; DHA, docosahexaenoic acid; Eo-HDP, early-onset hypertensive disorders of pregnancy; EPA, eicosapentaenoic acid; GDM, gestational diabetes mellitus; HDP, hypertensive disorders of pregnancy; Lo-HDP, late-onset hypertensive disorders of pregnancy.
